# Immunohistochemical Expression of M30, BCL-2, BAX, and Ki-67 in Pancreatic Ductal Adenocarcinoma: Association with Clinicopathological Features

**DOI:** 10.3390/diagnostics16203426

**Published:** 2026-10-21

**Authors:** Betül Demirciler Yavaş, Sezgin Yılmaz

**Affiliations:** 1 Private Practice, Traditional and Complementary Treatment Center, 03030 Afyonkarahisar, Türkiye; 2 Department of General Surgery, Faculty of Medicine, Afyonkarahisar Health Sciences University, 03200 Afyonkarahisar, Türkiye

**Keywords:** pancreatic ductal adenocarcinoma, immunohistochemistry, apoptosis, M30, BCL-2, BAX, Ki-67, clinicopathological features

## Abstract

**Background:** Pancreatic ductal adenocarcinoma (PDAC) is an aggressive malignancy with limited survival despite advances in diagnosis and treatment. Dysregulation of apoptosis and tumor cell proliferation is a fundamental feature of cancer biology. M30, BCL-2, and BAX reflect different aspects of apoptotic activity and regulation, whereas Ki-67 is a widely used marker of cellular proliferation. However, the clinicopathological significance of these markers in PDAC remains incompletely characterized. This study aimed to evaluate the immunohistochemical expression of M30, BCL-2, BAX, and Ki-67 in PDAC and to investigate their associations with established clinicopathological features. **Methods:** This retrospective study included 50 patients with surgically resected PDAC treated at Afyon Kocatepe University Medical Faculty Hospital between January 2003 and May 2015. Archived tumor specimens were evaluated by immunohistochemistry for M30, BCL-2, BAX, and Ki-67. M30 and Ki-67 expression were quantified according to the percentage of positive tumor cells, while BCL-2 and BAX expression were assessed using semiquantitative staining scores based on staining intensity and the proportion of positive tumor cells. Immunohistochemical findings were compared with sex, tumor differentiation, tumor size, lymphovascular invasion, perineural invasion, surgical margin status, tumor stage, lymph node metastasis, and clinical stage. **Results:** The study cohort comprised 30 male and 20 female patients, with a mean age of 62.12 ± 10.93 years. M30 expression was detected in 39 (78%) cases, whereas Ki-67 expression ranged from 10% to 85% of tumor cells. BCL-2 and BAX expression showed variable staining patterns. None of the four markers demonstrated a statistically significant association with tumor differentiation, tumor size, lymphovascular invasion, perineural invasion, surgical margin status, tumor stage, lymph node metastasis, or clinical stage. **Conclusions:** In this cohort of surgically resected PDAC, immunohistochemical expression of M30, BCL-2, BAX, and Ki-67 was not significantly associated with conventional clinicopathological indicators of tumor aggressiveness. These findings indicate that immunohistochemical assessment of individual apoptosis- and proliferation-related markers may not adequately reflect tumor aggressiveness or clinicopathological heterogeneity in PDAC. Further studies incorporating larger cohorts, standardized immunohistochemical assessment, and clinical outcome data are warranted to clarify their potential biological and prognostic significance.

## 1. Introduction

Pancreatic ductal adenocarcinoma (PDAC) is one of the most aggressive and lethal malignancies of the gastrointestinal tract and represents the vast majority of pancreatic cancers [1]. Although pancreatic cancer accounts for a relatively small proportion of all malignant tumors, it remains a leading cause of cancer-related mortality worldwide due to its aggressive biological behavior, late clinical presentation, and limited therapeutic options [2]. Despite advances in surgical techniques, chemotherapy regimens, and supportive care, the long-term prognosis of patients with PDAC remains poor, with five-year survival rates remaining below 15% in most series [3]. The clinical outcome of PDAC is influenced by several established clinicopathological factors. Tumor stage, lymph node involvement, tumor size, histological grade, lymphovascular invasion, perineural invasion, surgical margin status, and extra-pancreatic extension have all been associated with disease progression and patient survival [4]. However, the biological mechanisms underlying the aggressive behavior of PDAC are complex and incompletely understood. Therefore, considerable attention has been directed toward identifying molecular markers that may provide additional information regarding tumor biology, prognostic stratification, and potential therapeutic targets [5]. In this context, acquiring adequate tissue specimens through advanced sampling techniques—such as endoscopic ultrasound-guided fine-needle aspiration (EUS-FNA)—plays a critical role in the accurate preoperative histological diagnosis, grading, and biomarker evaluation of solid pancreatic lesions [6,7]. However, the biological mechanisms underlying the aggressive behavior of PDAC are complex and incompletely understood. Therefore, considerable attention has been directed toward identifying molecular and microenvironmental markers that may provide additional information regarding tumor biology, prognostic stratification, and potential therapeutic targets [8,9]. For instance, recent studies have demonstrated that immune microenvironment markers—such as HCST expression for defining immune-hot versus immune-cold subtypes or PD-L1 expression in specific histopathological subtypes—may offer critical insights into biological aggressiveness and patient outcomes in pancreatic neoplasms. Deregulation of apoptosis and uncontrolled cellular proliferation are fundamental hallmarks of carcinogenesis [8]. Alterations in these processes contribute to tumor initiation, progression, metastatic potential, and resistance to treatment. Consequently, proteins involved in apoptotic signaling and cell-cycle regulation have been extensively investigated in various human malignancies [9]. M30 is a monoclonal antibody that recognizes a neo-epitope generated by caspase-mediated cleavage of cytokeratin 18 during the early stages of apoptosis [10]. Because cytokeratin 18 is predominantly expressed in epithelial cells, M30 serves as a specific marker of epithelial cell apoptosis. M30 expression can be evaluated immunohistochemically in tissue specimens and has also been measured in serum using enzyme-linked immunosorbent assay (ELISA)-based methods [11]. Previous studies have suggested that M30 expression may reflect apoptotic activity and may be associated with tumor behavior and prognosis in several epithelial malignancies, including colorectal and endometrial carcinomas [12]. Ki-67 is a well-established marker of cellular proliferation and is expressed during all active phases of the cell cycle (G1, S, G2, and M phases) but not during the resting phase (G0) [13]. The Ki-67 labeling index is widely used to estimate proliferative activity in neoplastic tissues and has been associated with aggressive biological behavior and unfavorable prognosis in numerous cancers [14,15,16]. Members of the B-cell lymphoma 2 (BCL-2) family play a central role in the regulation of apoptosis through the mitochondrial pathway [17]. BCL-2, the first identified apoptosis-regulating gene, functions primarily as an anti-apoptotic protein by inhibiting programmed cell death and promoting cellular survival [18]. Increased BCL-2 expression has been reported in several malignancies, including lymphomas, malignant melanoma, and lung carcinoma, suggesting a role in tumor development and progression [19]. In contrast, BCL-2-associated X protein (BAX) is a pro-apoptotic member of the same protein family and promotes apoptosis by facilitating mitochondrial membrane permeabilization and activation of downstream caspase pathways [20]. Reduced expression or functional inactivation of BAX has been associated with impaired apoptotic responses and adverse clinical outcomes in various malignancies, including breast and oral cavity carcinomas [21,22]. Although apoptosis- and proliferation-related biomarkers have been investigated in numerous cancer types, their clinicopathological significance in PDAC remains incompletely defined. A better understanding of the expression patterns of these markers may contribute to elucidating the biological behavior of pancreatic cancer and their potential role in prognostic assessment. Therefore, the aim of the present study was to evaluate the immunohistochemical expression of M30, BCL-2, BAX, and Ki-67 in surgically resected PDAC specimens and to investigate their associations with established clinicopathological prognostic parameters.

## 2. Materials and Methods

### 2.1. Study Design and Patient Selection

This retrospective study included 50 patients diagnosed with pancreatic ductal adenocarcinoma (PDAC) who underwent surgical resection at Afyonkarahisar Health Sciences University, Health Research and Application Center, between January 2003 and May 2015. Clinicopathological data were retrieved from the archives of the Department of Medical Pathology and hospital records. The evaluated parameters included patient sex, tumor differentiation, tumor size, lymphovascular invasion, perineural invasion, surgical margin status, lymph node metastasis, tumor stage, and clinical stage. Tumor staging was reassessed according to the 7th edition of the American Joint Committee on Cancer (AJCC) Cancer Staging Manual for pancreatic carcinoma. Hematoxylin and eosin (H&E)-stained slides were reviewed, and the most representative formalin-fixed paraffin-embedded tissue block from each case was selected for immunohistochemical analysis. The study protocol was reviewed and approved by the Clinical Research Ethics Committee of Afyonkarahisar Health Sciences University (approval date: 11 June 2015; approval number: 2015/09-244). The requirement for individual informed consent was formally waived by the ethics committee owing to the retrospective study design, the use of anonymized historical clinical records, and the reliance on archived tissue specimens. The study was conducted in strict accordance with the ethical principles outlined in the Declaration of Helsinki.

### 2.2. Immunohistochemistry

Four-micrometer-thick sections were cut from formalin-fixed paraffin-embedded (FFPE) tissue blocks and mounted on positively charged slides. Immunohistochemical staining was performed using an automated staining platform (Leica Bond-Max, Leica Biosystems, Nußloch, Germany) according to the manufacturer’s standardized protocols. The specific primary antibody clones, source manufacturers, dilutions, and heat-induced epitope retrieval (HIER) conditions utilized for M30, BCL-2, BAX, and Ki-67 are detailed in Table 1. Stained slides were independently examined using a light microscope (Eclipse E600, Nikon, Tokyo, Japan).

### 2.3. Evaluation of Immunohistochemical Staining

M30 Assessment: Staining was considered positive when more than 1% of tumor cells showed cytoplasmic immunoreactivity. This threshold was selected according to the predefined assessment criteria used in the immunohistochemical evaluation. The Ki-67 labeling index was expressed as the percentage of positively stained tumor cell nuclei among at least 1000 evaluated tumor cells. It should be emphasized that this cohort-derived median threshold (34%) was utilized strictly for sample-specific, exploratory categorical comparisons within this study and does not represent an internationally standardized or clinically validated prognostic cutoff point. BCL-2 staining was evaluated according to both staining intensity (0–3) and the percentage of positive tumor cells (0–4), and a total immunoreactivity score was calculated by multiplying these two components. For categorical analysis, cases were stratified into groups with scores ≤2 and >2. This categorization was based on the distribution of immunoreactivity scores in the present cohort and was used for exploratory statistical comparisons rather than as a clinically validated cutoff. BAX staining was evaluated using the same semiquantitative scoring system, with staining intensity scored from 0 to 3 and the percentage of positive tumor cells scored from 0 to 4. The total immunoreactivity score was calculated by multiplying the two component scores. For categorical analysis, cases were stratified into groups with scores ≤3 and >3. This categorization was based on the score distribution in the present cohort and was used for exploratory statistical comparisons rather than as a clinically validated cutoff.

Staining intensity was scored as follows: 0 = no staining; 1 = weak staining; 2 = moderate staining; 3 = strong staining. The percentage of positive tumor cells was scored as follows: 0 = no staining; 1 = <10%; 2 = 10–50%; 3 = 51–80%; 4 = >80%. A total immunoreactivity score (IRS) was obtained by multiplying the staining intensity score (0–3) by the percentage score (0–4), yielding a final score ranging from 0 to 12. This semiquantitative multiplication matrix is based on the established Remmele and Stegner immunoreactive score index, which is widely recognized and validated for evaluating immunohistochemical expression of apoptosis- and cell-cycle-related biomarkers in tumor tissues [17,23,24]. BAX Assessment: BAX expression was evaluated using the same semiquantitative scoring system applied for BCL-2. Cytoplasmic and membranous staining intensity and the percentage of positive tumor cells were assessed separately, and a total immunoreactivity score was calculated by multiplying these two parameters.

To minimize the potential confounding influence of intratumoral biological and spatial heterogeneity during visual assessment and manual counting, whole-tissue sections—rather than tissue microarrays—were evaluated, and representative hot-spot areas as well as diverse microscopic fields were systematically examined across each tumor. Nevertheless, manual estimation remains inherently subject to sampling variation caused by regional variations in staining intensity and positive cell distribution. To ensure evaluation reliability, immunohistochemical scoring was performed independently by experienced pathologists who were blinded to the clinicopathological outcomes. Interobserver agreement was formally assessed, demonstrating high concordance across evaluated markers.

### 2.4. Statistical Analysis

Statistical analyses were performed using IBM SPSS Statistics software (version 22.0; IBM Corp., Armonk, NY, USA). Continuous variables (M30 and Ki-67 percentages; BCL-2 and BAX total immunoreactivity scores) were evaluated using non-parametric methods due to non-normal data distributions. The assumptions for non-parametric testing were met prior to analysis: for the *Mann–Whitney U* test, independent observations, continuous dependent variables, and a binary grouping variable were confirmed; for the chi-square (χ^2^) test of independence, independent categorical observations and mutually exclusive categories were verified, with minimum expected cell counts confirmed (≥5 in at least 80% of cells). Continuous variable comparisons across binary subgroups were conducted via the *Mann–Whitney U* test using the Analyze > Nonparametric Tests > Legacy Dialogs > 2 Independent Samples module in SPSS (with test type set to *Mann–Whitney U* and exact/asymptotic *p*-values computed). Categorical variable associations were evaluated using the chi-square test via the Analyze > Descriptive Statistics > Crosstabs module (selecting Chi-square under Statistics and displaying Observed counts alongside Row/Column Percentages under Cells). *Fisher’s* exact test was applied when expected cell counts fell below 5. All statistical tests were two-tailed, and a *p*-value <0.05 was considered statistically significant. Given the fixed sample size of 50 resected PDAC cases, post hoc statistical power calculations indicated that the study had sufficient power (≥80%) to detect medium-to-large effect sizes but was limited in its statistical power to identify subtle or minor associations between biomarker expression and clinicopathological variables.

## 3. Results

### 3.1. Clinicopathological Characteristics

A total of 50 patients with pancreatic ductal adenocarcinoma (PDAC) were included in the study. Patient age ranged from 21 to 81 years, with a mean age of 62.12 ± 10.93 years. The cohort consisted of 30 (60%) males and 20 (40%) females. Histopathological examination revealed 12 (24%) well-differentiated, 29 (58%) moderately differentiated, and 9 (18%) poorly differentiated tumors. Tumor size ranged from 1 to 8.5 cm, with a mean diameter of 3.78 ± 1.92 cm. Lymphovascular invasion was identified in 16 (32%) cases, whereas perineural invasion was present in 26 (52%) cases. Surgical margin positivity was observed in 8 (16%) patients. According to the AJCC TNM staging system, 6 (12%) tumors were classified as pT1, 13 (26%) as pT2, and 31 (62%) as pT3. Lymph node metastasis was detected in 23 (46%) patients, while 27 (54%) patients had no lymph node involvement. Clinical stage I disease was present in 13 (26%) patients, whereas 37 (74%) patients had stage II disease.

### 3.2. Immunohistochemical Findings

**Figure 1:**
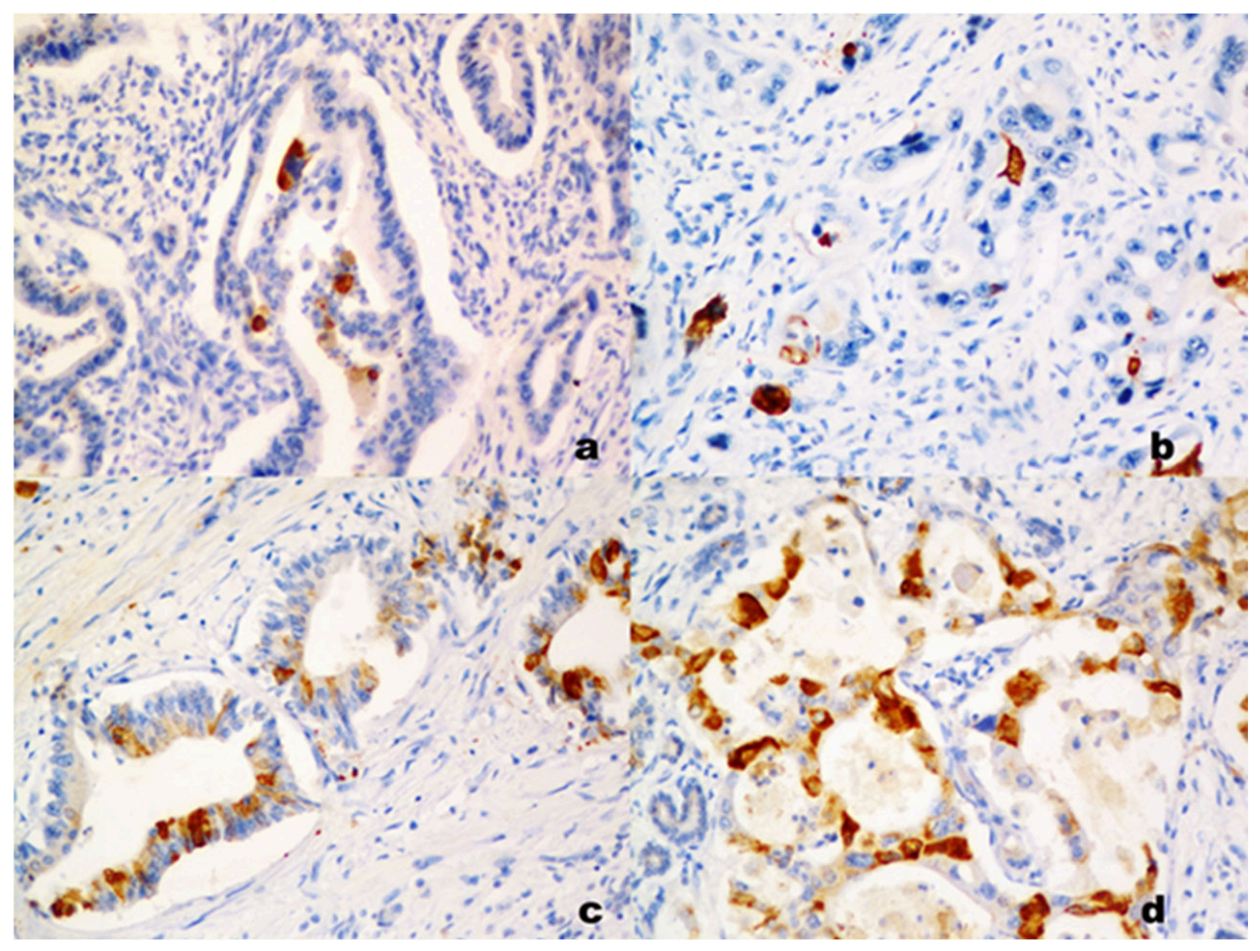
Representative immunohistochemical staining patterns of M30 in pancreatic ductal adenocarcinoma tissues: (**a**) absence of M30 immunoreactivity in tumor cells (score 0); (**b**) focal M30 positivity in a limited number of neoplastic cells (score 1); (**c**) moderate M30 expression with increased numbers of positively stained tumor cells (score 2); and (**d**) strong and diffuse M30 immunoreactivity in a high proportion of tumor cells (score 3). Positive staining is identified by brown cytoplasmic immunoreactivity. Original magnification, ×400.

**Figure 2:**
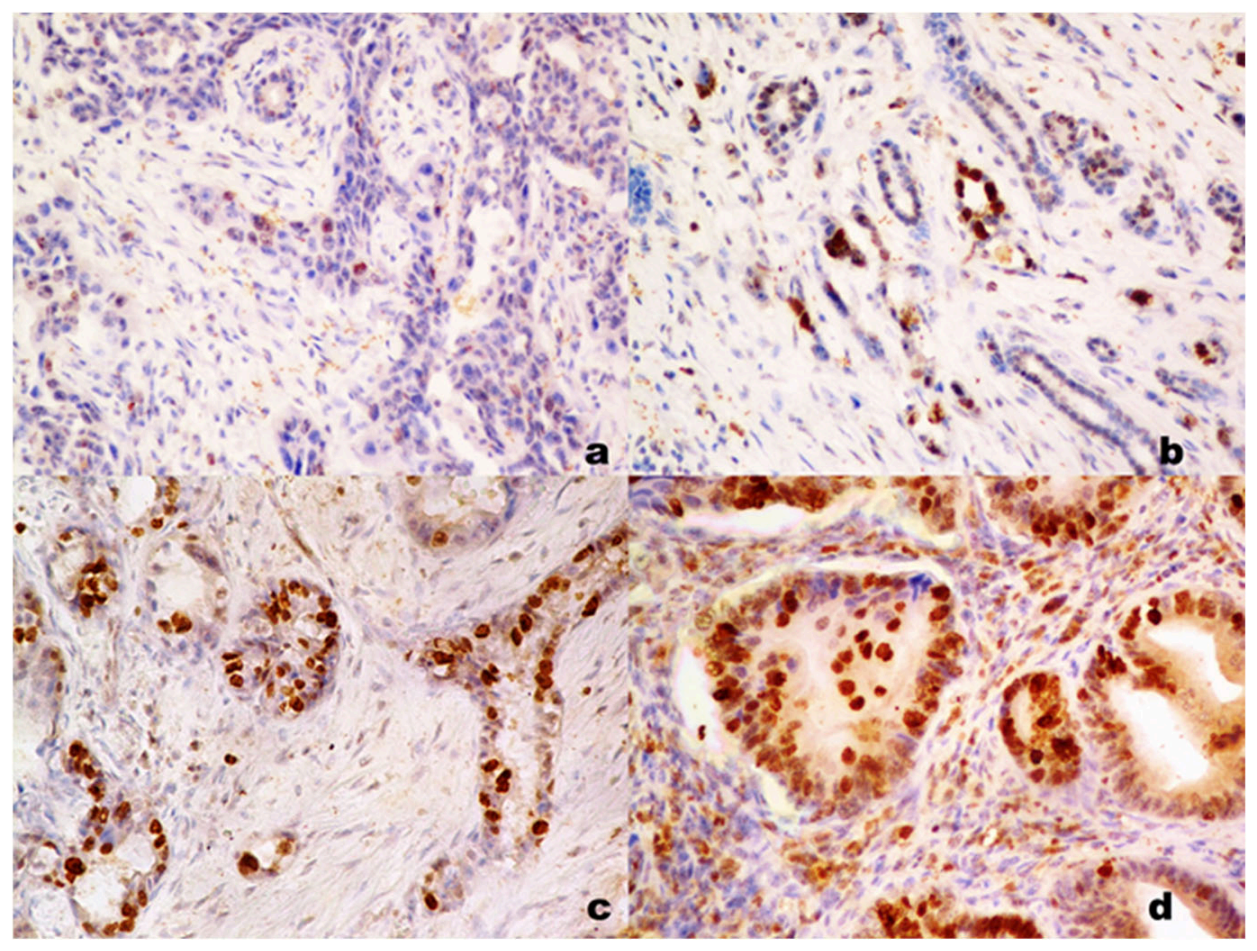
Representative immunohistochemical expression of Ki-67 in pancreatic ductal adenocarcinoma: (**a**) negative Ki-67 immunoreactivity (score 0); (**b**) weak cytoplasmic staining in a limited proportion of tumor cells (score 1–2); (**c**) moderate Ki-67 expression (score 3–4); and (**d**) strong and diffuse cytoplasmic BCL-2 immunoreactivity in the majority of neoplastic cells (score 5–6). Positive staining is identified by brown cytoplasmic staining of tumor cells. Original magnification, ×400.

**Figure 3:**
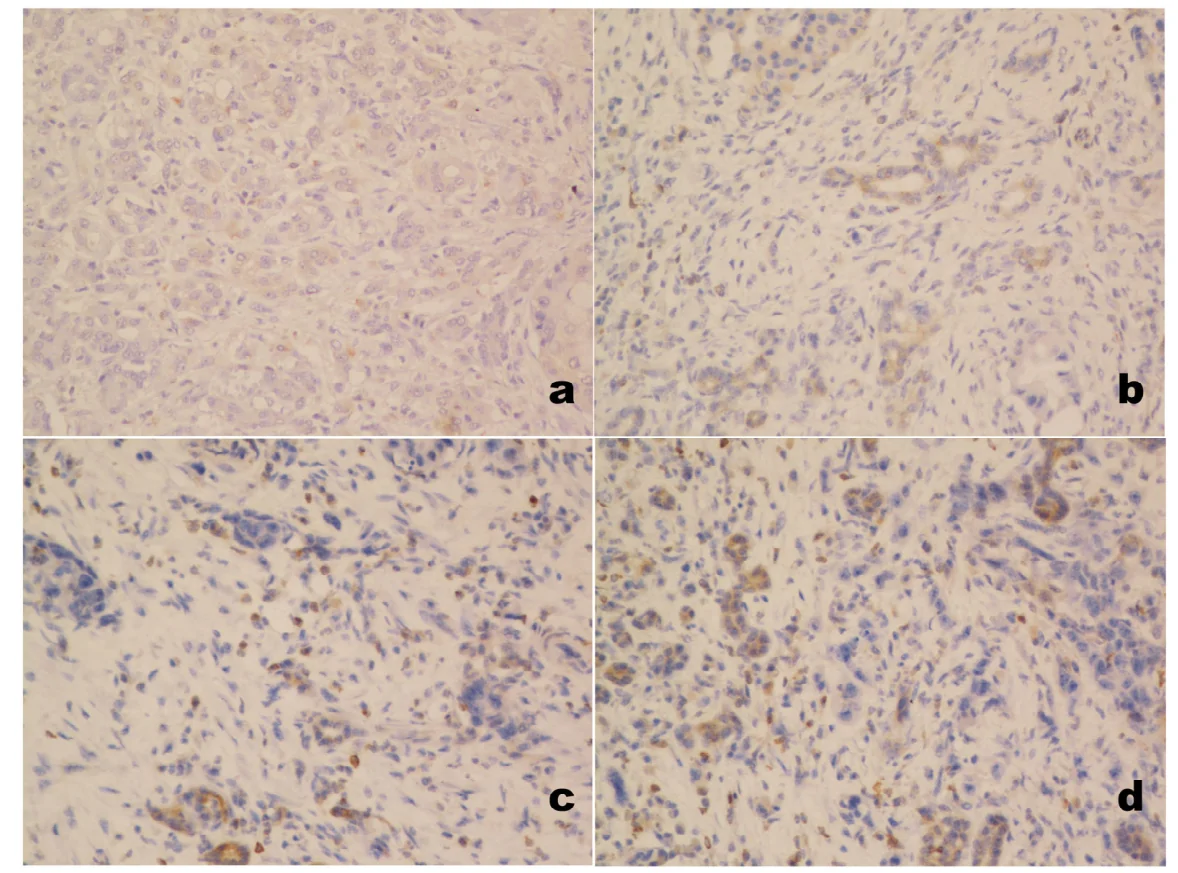
Representative immunohistochemical expression of BCL-2 in pancreatic ductal adenocarcinoma: (**a**) negative BCL-2 immunoreactivity (score 0); (**b**) weak cytoplasmic staining in a limited proportion of tumor cells (score 1–2); (**c**) moderate BCL-2 expression (score 3–4); and (**d**) strong and diffuse cytoplasmic BCL-2 immunoreactivity in the majority of neoplastic cells (score 5–6). Positive staining is identified by brown cytoplasmic staining of tumor cells. Original magnification, ×400.

**Figure 4:**
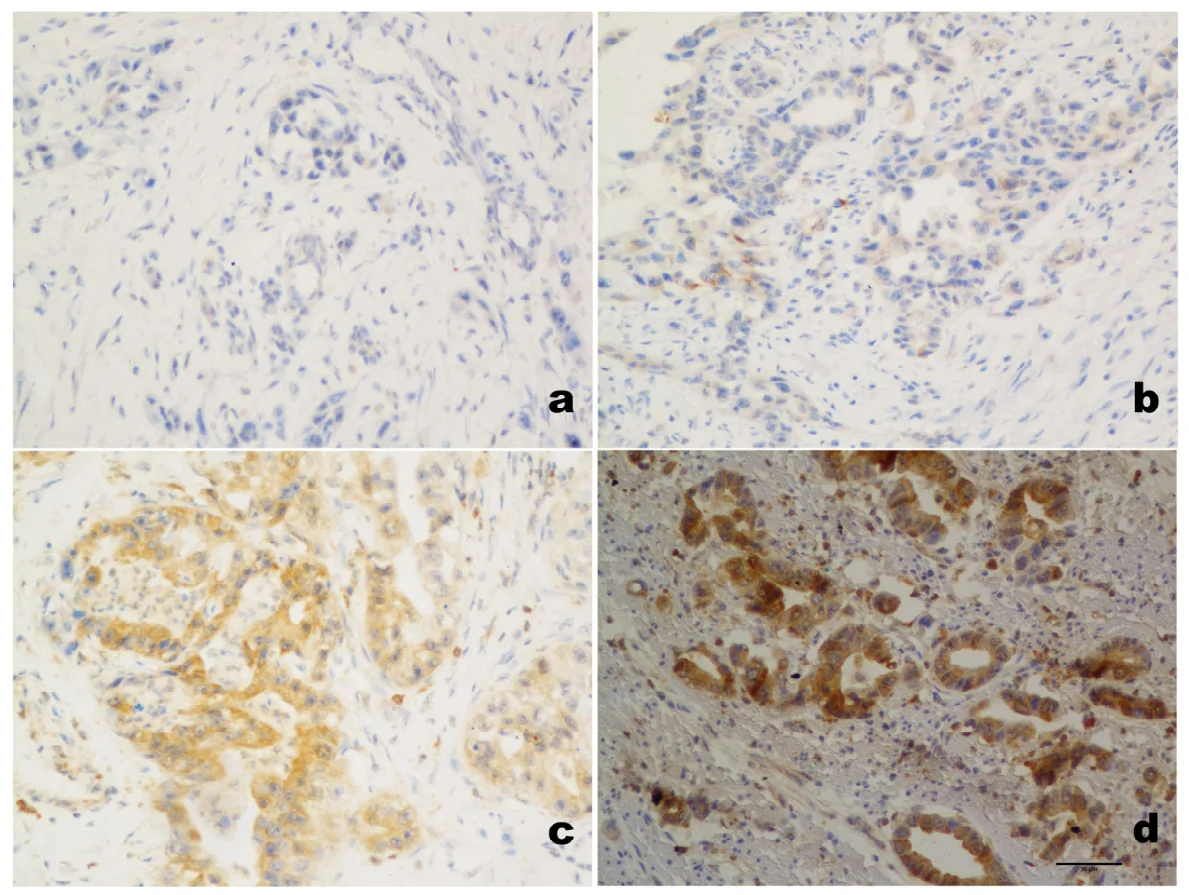
Representative immunohistochemical expression of BAX in pancreatic ductal adenocarcinoma: (**a**) negative BAX staining (intensity score 0); (**b**) weak cytoplasmic BAX immunoreactivity (intensity score 1); (**c**) moderate cytoplasmic BAX expression (intensity score 2); and (**d**) strong cytoplasmic BAX expression (intensity score 3). Positive tumor cells exhibit brown cytoplasmic immunostaining. Original magnification, ×400.

M30 immunoreactivity was detected in 39 (78%) of 50 PDAC cases, whereas 11 (22%) cases showed no detectable staining. Cases were classified as M30-positive and M30-negative according to the predefined cutoff value. Comparative analyses demonstrated no statistically significant associations between M30 expression status and sex, tumor differentiation, tumor size, lymphovascular invasion, perineural invasion, surgical margin status, tumor stage, lymph node metastasis, or clinical stage (*p* > 0.05) (Figure 1 and Table 2). Ki-67 immunostaining was observed in all tumor specimens. The proportion of positively stained tumor cells ranged from 10% to 85%. Based on the median Ki-67 labeling index, cases were categorized into low-expression (≤34%) and high-expression (>34%) groups. No significant differences were identified between low and high Ki-67 expression groups with respect to sex, tumor differentiation, tumor size, lymphovascular invasion, perineural invasion, surgical margin status, tumor stage, lymph node metastasis, or clinical stage (*p* > 0.05) (Figure 2 and Table 3). Variable degrees of BCL-2 expression were observed among the examined PDAC specimens. Thirty-four (68%) cases demonstrated a BCL-2 immunoreactivity score of ≤2, whereas 16 (32%) cases had a score > 2. Comparison of clinicopathological characteristics between low and high BCL-2 expression groups revealed no statistically significant differences regarding sex, tumor differentiation, tumor size, lymphovascular invasion, perineural invasion, surgical margin status, tumor stage, lymph node metastasis, or clinical stage (*p* > 0.05) (Figure 3 and Table 4). BAX expression showed heterogeneous staining patterns among PDAC cases. Based on the mean staining score, 19 (38%) tumors were classified as having low BAX expression (score ≤ 3), whereas 31 (62%) tumors were classified as having high BAX expression (score > 3). No significant associations were found between BAX expression and any evaluated clinicopathological parameter, including sex, tumor differentiation, tumor size, lymphovascular invasion, perineural invasion, surgical margin status, tumor stage, lymph node metastasis, or clinical stage (*p* > 0.05) (Figure 4 and Table 5). Overall, immunohistochemical expression levels of M30, Ki-67, BCL-2, and BAX were not significantly associated with conventional clinicopathological indicators of tumor aggressiveness in this cohort of surgically resected pancreatic ductal adenocarcinoma.

## 4. Discussion

Pancreatic ductal adenocarcinoma (PDAC) remains one of the most aggressive malignancies worldwide and continues to be associated with poor clinical outcomes despite advances in surgical techniques and systemic therapies [2]. The biological behavior of PDAC is characterized by rapid progression, early metastatic spread, and resistance to treatment, making the identification of reliable prognostic biomarkers an important area of research [1]. In the present study, we investigated the immunohistochemical expression of the proliferation marker Ki-67 and the apoptosis-related markers M30, BCL-2, and BAX in surgically resected PDAC specimens and evaluated their associations with established clinicopathological parameters. None of the examined markers demonstrated a significant relationship with tumor differentiation, tumor size, lymphovascular invasion, perineural invasion, surgical margin status, tumor stage, lymph node metastasis, or clinical stage. Cellular proliferation is a fundamental characteristic of malignant tumors, and Ki-67 is among the most widely used proliferation markers in diagnostic pathology [13]. Increased Ki-67 expression has been associated with aggressive tumor behavior and unfavorable prognosis in various malignancies [14,15,16]. However, studies evaluating the prognostic significance of Ki-67 in PDAC have yielded conflicting results. Regarding Ki-67, published studies have reported variable associations between Ki-67 expression and clinicopathological characteristics or outcomes in PDAC. Such discrepancies may partly reflect differences in study populations, sample sizes, tumor characteristics, and the methods used to assess Ki-67 expression. In particular, differences in antibody clones, staining and tissue-processing procedures, methods of selecting tumor areas, and scoring approaches may influence the reported labeling indices. Furthermore, the use of different or study-specific cutoff values may substantially affect categorical comparisons and limit direct comparability between studies. Therefore, the lack of significant associations observed in the present study should be interpreted cautiously and may reflect, at least in part, methodological and biological heterogeneity rather than the absence of a clinically relevant relationship. In our study, classifying cases into low- and high-expression groups based on a sample-specific median value of 34% inherently reflects the specific distribution of our 50-patient cohort. Reliance on sample-dependent medians rather than universally accepted cutoffs hampers direct cross-study comparisons and restricts external generalizability. Until standardized, clinically validated cutoff values for Ki-67 are established in PDAC, findings derived from sample-specific thresholds must be interpreted as exploratory. While some investigators reported an association between elevated Ki-67 expression and adverse clinical outcomes, others failed to identify significant correlations with pathological characteristics or patient survival [25,26,27]. In the present study, Ki-67 expression was detected in all tumor samples, with labeling indices ranging from 10% to 85%. Nevertheless, no statistically significant association was observed between Ki-67 expression levels and any of the evaluated clinicopathological parameters. These findings support previous reports suggesting that proliferative activity alone may not adequately reflect the biological complexity of PDAC. Apoptosis plays a critical role in maintaining tissue homeostasis, and disruption of apoptotic signaling pathways contributes to carcinogenesis and tumor progression [7]. The BCL-2 family of proteins constitutes a major regulatory system of the intrinsic apoptotic pathway and includes both anti-apoptotic and pro-apoptotic members [17]. BCL-2 functions primarily as an anti-apoptotic protein that promotes cell survival, whereas BAX facilitates apoptosis through mitochondrial outer membrane permeabilization and activation of downstream apoptotic mechanisms [18,20]. Altered expression of BCL-2 family proteins has been reported in numerous malignancies and has frequently been associated with tumor progression, treatment response, and prognosis [17,19]. However, studies focusing specifically on PDAC have produced inconsistent findings regarding the clinicopathological significance of BCL-2 and BAX expression [28,29,30,31]. In our study, neither BCL-2 nor BAX expression showed a significant association with any of the examined pathological variables. These findings are largely consistent with previous investigations reporting limited prognostic utility of individual BCL-2 family proteins in pancreatic carcinoma [23,29,32]. Similarly, studies investigating BCL-2 and BAX expression in PDAC have yielded inconsistent results regarding their associations with tumor differentiation, stage, metastasis, and prognosis. Differences in patient selection, cohort size, disease stage distribution, and the biological characteristics of the tumors may contribute to these divergent findings. In addition, immunohistochemical variability, including antibody selection, tissue processing, staining interpretation, scoring systems, and the thresholds used to define positive or high expression, may influence the observed associations. The biological heterogeneity of PDAC may further contribute to differences between cohorts, as tumors with apparently similar clinicopathological features may exhibit distinct molecular and apoptotic profiles. In this context, the absence of significant clinicopathological associations for BCL-2 and BAX in our cohort should be regarded as a finding from an exploratory, relatively small cohort rather than evidence against a potential biological or prognostic role of these markers. Considering the complexity of apoptotic regulation in PDAC, it is likely that multiple interacting molecular pathways contribute to tumor behavior, thereby limiting the ability of single biomarkers to predict clinicopathological characteristics. Furthermore, evaluating individual apoptotic regulators in isolation overlooks the intricate functional balance between pro-apoptotic (e.g., BAX, M30) and anti-apoptotic (e.g., BCL-2) signaling pathways. Future studies employing standardized scoring strategies and focusing on functional ratios will be essential to better capture the dynamic apoptotic landscape of PDAC. M30 is a well-recognized marker of epithelial cell apoptosis that detects caspase-cleaved cytokeratin 18 fragments generated during the early stages of programmed cell death [10]. Compared with Ki-67 and BCL-2 family proteins, relatively few studies have evaluated the significance of M30 expression in pancreatic carcinoma [33,34]. Available data have generally reported inconsistent associations between M30 expression and clinicopathological characteristics or clinical outcomes [34,35,36]. Similarly, in the present study, M30 expression was not significantly associated with any of the evaluated prognostic parameters. These findings suggest that the level of apoptotic activity assessed by M30 immunohistochemistry may not directly correspond to conventional indicators of tumor aggressiveness in PDAC. The absence of significant associations between the investigated biomarkers and clinicopathological parameters may be explained by several factors. Consequently, the absence of statistically significant correlations across the evaluated clinicopathological parameters must be interpreted with caution. Due to the cohort size (*n* = 50), the study was primarily powered to detect moderate-to-large biological effects. Small to moderate trends may have remained undetected, and the findings should be regarded as exploratory observations within a single-center resected cohort rather than conclusive evidence against biomarker relevance. Importantly, the absence of statistically significant correlations in our study should not be interpreted as definitive proof that M30, BCL-2, BAX, and Ki-67 lack biological or prognostic relevance in PDAC. Small sample sizes substantially limit statistical power to detect subtle or modest biological associations, increasing the likelihood of Type II errors. Rather than relying solely on post hoc power assessments, future studies should focus on reporting robust effect size estimates along with 95% confidence intervals to better quantify the potential clinical significance of these biomarkers. PDAC is characterized by substantial molecular and biological heterogeneity, and individual immunohistochemical markers may be insufficient to reflect the complex mechanisms underlying tumor progression [1,5]. Furthermore, immunohistochemical evaluation provides only a static assessment of protein expression and may not accurately represent the dynamic balance between proliferation and apoptosis within the tumor microenvironment [5,17]. Therefore, the biological behavior of PDAC is likely determined by the interaction of multiple molecular pathways rather than the expression status of a single biomarker. Furthermore, because our evaluation focused exclusively on baseline clinicopathological parameters without incorporating survival or recurrence endpoints (such as overall survival, disease-free survival, or treatment-response data), we cannot definitively establish the prognostic value of M30, BCL-2, BAX, or Ki-67, nor can we conclude that they are clinically entirely uninformative. Future investigations incorporating longitudinal survival tracking and multivariable Cox proportional hazards regression models adjusted for established clinicopathological prognostic factors are essential to thoroughly validate or refute the prognostic utility of these biomarkers in PDAC. Several limitations of this study should be acknowledged. First, the retrospective design may have introduced selection bias. Additionally, the long enrollment timeframe (2003–2015) introduces potential confounding due to historical shifts in clinical practice. Over this 12-year period, surgical techniques, pathological margin assessment protocols, and systemic treatment regimens (such as the widespread introduction of neoadjuvant therapy and modern combination chemotherapy regimens) underwent significant evolution. Because treatment protocols were not uniform across the historical cohort, unmeasured therapeutic variations over time may have confounded the biological impact or observable expression patterns of the investigated biomarkers. Second, the relatively small sample size may have limited the statistical power to detect subtle associations. Third, survival and follow-up data were not available, preventing direct evaluation of the prognostic value of the investigated markers. Finally, molecular analyses were not performed, and the study relied exclusively on immunohistochemical assessment. Future studies involving larger patient cohorts, comprehensive molecular profiling, and survival analyses are needed to further elucidate the biological and clinical significance of apoptosis- and proliferation-related markers in PDAC. The lack of significant associations between individual IHC markers and clinicopathological parameters underscores the intricate molecular redundancy governing PDAC biology. The balance between apoptosis and proliferation is regulated by dynamic crosstalk involving the intrinsic mitochondrial apoptotic cascade, p53 signaling networks, and downstream KRAS-driven effector pathways. Evaluating individual markers like Ki-67, M30, BCL-2, or BAX in isolation provides only a static snapshot of complex, interconnected physiological processes. To enhance clinical relevance, future translational research should move beyond single-biomarker immunohistochemistry toward integrated multi-marker prognostic models and spatial transcriptomic profiling. Exploring functional ratios (such as the BAX/BCL-2 balance relative to apoptotic indices) in combination with key driver mutation statuses (e.g., KRAS, TP53, CDKN2A, SMAD4) may uncover distinct molecular subtypes with specific clinical behaviors. Additionally, functional in vitro and organoid model studies are warranted to evaluate how alterations in these apoptotic pathways directly influence chemoresistance and therapeutic vulnerability in PDAC.

## 5. Conclusions

In this cohort of patients with pancreatic ductal adenocarcinoma, M30, BCL-2, BAX, and Ki-67 expression did not show significant associations with the examined clinicopathological characteristics. These findings suggest that the investigated immunohistochemical markers did not reliably distinguish clinicopathological subgroups within the present study population. However, given the limited sample size, retrospective design, and cohort-specific assessment of biomarker expression, these findings should be interpreted cautiously. The clinical utility and generalizability of these markers in PDAC cannot be established from the present data alone and require further validation in larger, independent, and preferably prospective cohorts with standardized immunohistochemical assessment and clinical follow-up.

### Limitations

This study has several limitations that should be considered when interpreting the findings. First, the retrospective design and the historical patient recruitment window (2003–2015) introduce inherent selection bias and temporal confounding. Surgical resectability criteria, pathological margin definitions, and perioperative chemotherapy protocols evolved substantially over this 12-year period and differ from contemporary standards of care, which may limit the direct applicability of these findings to modern PDAC patient cohorts receiving current systemic therapies. Second, the study was conducted at a single center and included a relatively small cohort (*n* = 50), which substantially limits the statistical power to detect clinically meaningful associations or subtle biological trends. Consequently, non-significant *p*-values must not be construed as evidence of absence regarding the biological role of M30, BCL-2, BAX, or Ki-67. While post hoc power calculations were conducted, effect size estimates accompanied by 95% confidence intervals provide a more meaningful evaluation of uncertainty and clinical magnitude. Larger, prospective multicenter cohorts are essential to provide adequately powered analyses with precise effect estimates. Third, the complete absence of longitudinal follow-up and survival data—such as overall survival, disease-free survival, recurrence patterns, or treatment-response metrics—precludes any evaluation of the true prognostic or predictive value of M30, BCL-2, BAX, and Ki-67. Consequently, our findings based solely on cross-sectional baseline parameters cannot establish their true prognostic capacity, nor do they permit the conclusion that these markers are clinically uninformative. Prospective studies incorporating comprehensive survival analyses and multivariable Cox regression models adjusted for standard clinical risk factors will be mandatory to definitively ascertain their prognostic relevance. Consequently, negative correlations with baseline static clinicopathological parameters cannot rule out potential associations with overall survival (OS), disease-free survival (DFS), or post-chemotherapy recurrence patterns. Future prospective studies with comprehensive longitudinal tracking are mandatory to establish whether these biomarkers hold clinical utility in survival prediction. Additionally, manual visual scoring of full-section slides may be confounded by intratumoral spatial heterogeneity, as focal variations in biomarker expression across different tumor regions can introduce sampling variability compared with automated whole-slide digital image analysis. Furthermore, the categorization of Ki-67 expression using a cohort-specific median threshold rather than externally validated diagnostic cutoffs restricts direct comparability with external populations and limits the clinical reproducibility of the stratified groupings. In addition, while scoring was conducted independently, the lack of formal multi-observer concordance testing limits the assessment of interobserver reproducibility. The reliance on cohort-derived cutoffs (such as 34% for Ki-67 and specific immunoreactivity thresholds for BCL-2 and BAX) rather than internationally standardized cutoffs further constrains external reproducibility and cross-study comparability. A more standardized assessment strategy—incorporating automated digital image analysis and evaluation of the functional balance between pro- and anti-apoptotic proteins—is warranted to enhance biological interpretation. The study focused exclusively on immunohistochemical assessment and did not incorporate molecular analyses. Furthermore, while our study evaluated biomarkers on surgically resected specimens, preoperative biomarker and grading assessments relying on tissue sampling from endoscopic ultrasound (EUS) have gained significant clinical interest. Considering the proven reliability and optimization of EUS-guided sampling methods in solid pancreatic lesions and neoplasms [6,7], future translational studies investigating these biomarkers in preoperative biopsy materials may provide valuable early predictive insights. Therefore, potential relationships between protein expression and underlying genetic or molecular alterations could not be investigated. Furthermore, immunohistochemical evaluation provides a static representation of protein expression at a single time point and may not fully reflect the dynamic biological processes of apoptosis and cellular proliferation occurring during tumor progression. Crucially, evaluating biomarker expression solely against static clinicopathological parameters (such as tumor size or lymph node status) provides limited clinical utility in predicting true biological aggressiveness. Static pathological features represent a single snapshot in time and do not necessarily capture longitudinal disease dynamics or patient treatment responses. Without survival outcomes—such as overall survival (OS) or disease-free survival (DFS)—it remains unclear whether these apoptosis and proliferation markers exert subtle prognostic effects that manifest independent of conventional staging. Therefore, while our cross-sectional findings show no correlation with static pathological indicators, definitive conclusions regarding prognostic value require integration with long-term patient follow-up data. Finally, because pancreatic ductal adenocarcinoma is a molecularly heterogeneous disease, the prognostic impact of individual biomarkers may be limited when evaluated in isolation. These limitations highlight the need for larger multicenter studies incorporating survival analyses, molecular profiling, and standardized immunohistochemical assessment methods to better define the biological and clinical significance of apoptosis- and proliferation-related biomarkers in pancreatic ductal adenocarcinoma.

## Figures and Tables

**Table 1:** Primary antibodies and immunohistochemical staining protocol parameters.

Target Antigen	Primary Antibody Clone	Manufacturer	Dilution	Antigen Retrieval (HIER)/Buffer	Incubation Time/Temp.
*M30*	Clone M30 (Mouse Monoclonal)	Roche Diagnostics/Formator	1:100	Citrate Buffer (pH 6.0), 95°, 20 min	30 min, Room Temp.
*BCL-2*	Clone 124 (Mouse Monoclonal)	Dako/Agilent	1:50	EDTA Buffer (pH 9.0), 98°, 20 min	30 min, Room Temp.
*BAX*	Clone 2D2 (Mouse Monoclonal)	Santa Cruz Biotechnology	1:100	Citrate Buffer (pH 6.0), 95°, 20 min	45 min, Room Temp.
*Ki-67*	Clone MIB-1 (Mouse Monoclonal)	Dako/Agilent	1:100	EDTA Buffer (pH 9.0), 98°, 20 min	30 min, Room Temp.

**Table 2:** Comparison of clinicopathological characteristics according to M30 expression status in pancreatic ductal adenocarcinoma.

Variable		M30 (+) *n* = 39 (78%)	M30 (−) *n* = 11 (22%)	*p*
Gender	Female	15 (38.5%)	5 (45.5%)	0.73
	Male	24 (61.5%)	6 (54.5%)	
Differentiation	Well–moderate	32 (82.1%)	9 (81.8%)	0.98
	Poor	7 (17.9%)	2 (18.2%)	
Tumor diameter	≤3.78 cm	22 (56.4%)	8 (72.7%)	0.48
	>3.78 cm	17 (43.6%)	3 (27.3%)	
Lymphovascular invasion	(−)	27 (69.2%)	7 (63.6%)	0.72
	(+)	12 (30.8%)	4 (36.4%)	
Perineural invasion	(−)	20 (51.3%)	4 (36.4%)	0.38
	(+)	19 (48.7%)	7 (63.6%)	
Surgical margin status	(−)	34 (87.2%)	8 (72.7%)	0.35
	(+)	5 (12.8%)	3 (27.3%)	
Clinical stage	Stage 1	12 (30.8%)	1 (9.1%)	0.24
	Stage 2	27 (69.2%)	10 (90.9%)	
Tumor stage	T1–T2	17 (43.6%)	2 (18.2%)	0.17
	T3	22 (56.4%)	9 (81.8%)	
Lymph node metastasis	N0	19 (48.7%)	4 (36.4%)	0.46
	N1	20 (51.3%)	7 (63.6%)	

**Table 3:** Comparison of clinicopathological characteristics according to Ki-67 expression level in pancreatic ductal adenocarcinoma.

Variable		≤34% Ki-67 *n* = 26 (52%)	>34% Ki-67 *n* = 26 (52%)	*p*
Gender	Female	10 (38.5%)	10 (41.7%)	0.81
	Male	16 (61.5%)	14 (58.3%)	
Differentiation	Well–moderate	20 (76.9%)	21 (87.5%)	0.46
	Poor	6 (23.1%)	3 (12.5%)	
Tumor diameter	≤3.78 cm	17 (65.4%)	13 (54.2%)	0.41
	>3.78 cm	9 (34.6%)	11 (45.8%)	
Lymphovascular invasion	(−)	17 (65.4%)	17 (70.8%)	0.68
	(+)	9 (34.6%)	7 (29.2%)	
Perineural invasion	(−)	12 (46.2%)	12 (50%)	0.78
	(+)	14 (53.8%)	19 (79.2%)	
Surgical margin status	(−)	23 (88.5%)	5 (20.8%)	0.45
	(+)	3 (11.5%)	3 (27.3%)	
Clinical stage	Stage 1	6 (23.1%)	7 (29.2%)	0.62
	Stage 2	20 (76.9%)	17 (70.8%)	
Tumor stage	T1–T2	9 (34.6%)	10 (41.7%)	0.6
	T3	17 (65.4%)	14 (58.3%)	
Lymph node metastasis	N0	11 (42.3%)	12 (50%)	0.58
	N1	15 (57.7%)	12 (50%)	

**Table 4:** Comparison of clinicopathological characteristics according to BCL-2 expression level in pancreatic ductal adenocarcinoma.

Variable		≤Score 2 BCL-2 *n* = 34 (68%)	>Score 2 BCL-2 *n* = 16 (32%)	*p*
Gender	Female	15 (44.1%)	5 (31.3%)	0.38
	Male	19 (55.9%)	11 (68.8%)	
Differentiation	Well–moderate	27 (79.4%)	14 (87.5%)	0.69
	Poor	7 (20.6%)	2 (12.5%)	
Tumor diameter	≤3.78 cm	22 (64.7%)	8 (50%)	0.32
	>3.78 cm	12 (35.3%)	8 (50%)	
Lymphovascular invasion	(−)	24 (70.6%)	10 (62.5%)	0.74
	(+)	10 (29.4%)	6 (37.5%)	
Perineural invasion	(−)	15 (44.1%)	9 (56.3%)	0.42
	(+)	19 (55.9%)	7 (43.8%)	
Surgical margin status	(−)	29 (85.3%)	13 (81.3%)	0.69
	(+)	5 (14.7%)	3 (18.8%)	
Clinical stage	Stage 1	8 (23.5%)	5 (31.3%)	0.73
	Stage 2	26 (76.5%)	11 (68.8%)	
Tumor stage	T1–T2	13 (38.2%)	6 (37.5%)	0.96
	T3	21 (61.8%)	10 (62.5%)	
Lymph node metastasis	N0	16 (47.1%)	7 (43.8%)	0.82
	N1	18 (52.9%)	9 (56.3%)	

**Table 5:** Comparison of clinicopathological characteristics according to BAX expression level in pancreatic ductal adenocarcinoma.

Variable		≤Score 3 BAX *n* = 19 (38%)	>Score 3 BAX *n* = 31 (62%)	*p*
Gender	Female	8 (42.1%)	12 (38.7%)	0.81
	Male	11 (57.9%)	19 (61.3%)	
Differentiation	Well–moderate	14 (73.7%)	27 (87.1%)	0.27
	Poor	5 (26.3%)	4 (12.9%)	
Tumor diameter	≤3.78 cm	12 (63.2%)	18 (58.1%)	0.72
	>3.78 cm	7 (36.8%)	13 (41.9%)	
Lymphovascular invasion	(−)	14 (73.7%)	20 (64.5%)	0.50
	(+)	5 (26.3%)	11 (35.5%)	
Perineural invasion	(−)	11 (57.9%)	13 (41.9%)	0.27
	(+)	8 (42.1%)	18 (58.1%)	
Surgical margin status	(−)	16 (84.2%)	26 (83.9%)	1.00
	(+)	3 (15.8%)	5 (16.1%)	
Clinical stage	Stage 1	5 (26.3%)	8 (25.8%)	1.00
	Stage 2	14 (73.7%)	23 (74.2%)	
Tumor stage	T1–T2	8 (42.1%)	11 (35.5%)	0.64
	T3	11 (57.9%)	20 (64.5%)	
Lymph node metastasis	N0	10 (52.6%)	13 (41.9%)	0.46
	N1	9 (47.4%)	18 (58.1%)	

## Data Availability

The data that support the findings of this study are available from the corresponding author upon reasonable request. The data are not publicly available due to ethical restrictions.
